# Evaluating the efficacy of different doses of tocilizumab in treating critically ill COVID–19 patients: a single–center retrospective cohort study

**DOI:** 10.3389/fphar.2025.1571372

**Published:** 2025-07-24

**Authors:** Jie Gao, Wenjing Zhang, Yaru Ding, Xinru Peng, Jing Wang, Yuting Li, Jing Gao, Jie Cheng, Wei Zhou, Shuxiang Zhang

**Affiliations:** ^1^ Department of Respiratory Medicine, First Clinical Medical College, Ningxia Medical University, Yinchuan, China; ^2^Department of Respiratory and Critical Care Medicine, General Hospital of Ningxia Medical University, Yinchuan, China; ^3^Department of Internal Medicine, Ningxia Yiyang Hospital, Yinchuan, China; ^4^Department of Respiratory Medicine, People’s Hospital of Ningxia Hui Autonomous Region, Ningxia Medical University, Yinchuan, China

**Keywords:** COVID-19, SARS-CoV-2, tocilizumab, IL-6, crp

## Abstract

**Objectives:**

To evaluate the therapeutic efficacy of different doses of tocilizumab (TCZ) in patients with severe or critical COVID–19.

**Methods:**

In this single–center retrospective cohort study conducted from January 2023 to January 2024, 56 hospitalized patients with severe or critical COVID–19 who received TCZ were included. Patients were categorized into three groups based on the number of TCZ doses administered: one dose (n = 16), two doses (n = 32), and three doses (n = 8). The primary outcomes were in–hospital mortality and 30–day mortality following the first dose. Secondary outcomes included changes in inflammatory marker levels, length of hospital stay, duration of mechanical ventilation, and incidence of complications during hospitalization.

**Results:**

After adjusting for potential confounders, there were no statistically significant differences in 30–day mortality (one dose vs. two doses HR 0.39; 95% CI, 0.15–1.04; P = 0.060 and one dose vs. three doses HR 0.27; 95% CI, 0.06–1.07; P = 0.067) or in–hospital mortality (one dose vs. two doses HR 0.65; 95% CI, 0.35–1.25; P = 0.090 and one dose vs. three doses HR 0.70; 95% CI, 0.40–1.50; P = 0.300) among the three groups. However, all groups showed a favorable response in inflammatory markers. Interleukin–6 (IL–6) levels initially increased after TCZ administration but subsequently declined in a fluctuating pattern. C–reactive protein (CRP) levels decreased consistently across all groups, while procalcitonin showed a modest decline. The number of TCZ doses had no significant impact on length of hospital stay, duration of mechanical ventilation, or the incidence of complications such as respiratory failure requiring mechanical ventilation, heart failure, secondary infections, thrombotic/embolic events, transaminase elevation, neutropenia, GI perforation/Haemorrhage, or acute kidney injury.

**Conclusion:**

Administering additional doses of TCZ beyond the initial dose was not associated with further reductions in mortality or improvements in other major clinical outcomes in patients with severe or critical COVID–19.

## 1 Introduction

Since its first outbreak in late 2019, Coronavirus Disease 2019 (COVID–19) has rapidly developed into a major global public health crisis. Caused by Severe Acute Respiratory Syndrome Coronavirus 2 (SARS–CoV–2), the infection can lead to pneumonia and, in severe cases, progress to Acute Respiratory Distress Syndrome (ARDS) and Multiple Organ Dysfunction Syndrome (MODS) (Hu et al., 2021). As patients deteriorate into stages involving ARDS, sepsis, and MODS, this phase is often characterized as cytokine storm or cytokine release syndrome (CRS). Although the virus itself may no longer be the primary driver at this stage, immune dysregulation, triggered by multiple underlying factors, leads to systemic inflammation and life–threatening multiorgan failure due to hyperactivation of the immune system. Cytokine storms have been directly associated with disease severity, morbidity, and mortality (Oran and Topol, 2021). As a result, therapeutic agents targeting interleukin–6 (IL–6) have been evaluated in several studies for their potential benefit in critically ill patients (Hermine et al., 2021; Salama et al., 2021).

Tocilizumab (TCZ) is a recombinant humanized IgG1 monoclonal antibody targeting the interleukin–6 receptor (IL–6R), originally developed for the treatment of certain rheumatologic conditions and later approved for use in patients with COVID–19. By blocking IL–6 signaling, TCZ is also used to manage cytokine storm induced by chimeric antigen receptor T–cell (CAR–T) therapy (Mahallawi et al., 2018). It acts as a competitive antagonist of both soluble and membrane–bound IL–6 receptors, thereby inhibiting both the cis–and trans–signaling pathways (Jones et al., 2005). As a consequence of this inhibition, serum IL–6 levels often increase following TCZ administration. This elevation is thought to reflect ongoing endogenous IL–6 production and underlying inflammatory activity, rather than diminished efficacy of the drug (Nishimoto et al., 2008). C–reactive protein (CRP) is commonly used as a surrogate marker to assess IL–6 bioactivity, as it correlates more reliably with the anti–inflammatory effects of IL–6 inhibitors, particularly in patients with severe disease. Moreover, clinical studies have shown that IL–6R inhibitors demonstrate similar safety and efficacy profiles compared to direct IL–6 inhibitors (Kaly and Rosner, 2012). Reducing IL–6 activity may lead to improved outcomes in patients with SARS–CoV–2 infection, as IL–6 plays a central role in both the pathophysiology of the disease and the severity of cytokine storm.

In previous studies, patients typically received an initial single dose of TCZ, with a second dose administered 8–72 h later if clinical improvement was not observed (Gordon et al., 2021; Horby et al., 2021; Veiga et al., 2021). These studies primarily focused on comparing TCZ treatment with standard care or placebo. However, in some cases, patients who received two full doses of TCZ showed limited clinical improvement, with persistent symptoms such as fever, dyspnea, poor oxygenation indices, and inflammatory infiltrates on lung imaging. At the same time, a severe cytokine storm driven by immune overactivation often remained active, continuing to inflict systemic damage. In such challenging scenarios, clinicians may cautiously consider administering a third dose of TCZ based on the patient’s overall condition, underlying comorbidities, current levels of inflammatory markers, and organ function status. Despite these considerations, there has been no comprehensive or systematic investigation into the potential benefits of different dosing regimens of TCZ on clinical outcomes, mortality, or laboratory indicators.

## 2 Materials and methods

### 2.1 Study design and patient recruitment

This single–center retrospective cohort study was conducted at the People’s Hospital of Ningxia Hui Autonomous Region and aimed to analyze the clinical characteristics of patients with severe or critical COVID–19 who were treated with TCZ between January 1, 2023, and January 1, 2024. A total of 56 patients were included based on the following criteria: age ≥18 years; confirmed SARS–CoV–2 infection by reverse transcription polymerase chain reaction (RT–PCR) from a nasopharyngeal or oropharyngeal swab; diagnosis of severe or critical illness according to national clinical guidelines (PRC and Medicine, 2023); and receipt of at least one dose of TCZ during hospitalization. Exclusion criteria included death within 48 h of hospital admission, prior treatment with TCZ before admission, or known allergy to any component of TCZ. All patients were followed until either hospital discharge or in–hospital death, whichever occurred first.

### 2.2 Treatment regimens

Written informed consent was obtained from patients or their accompanying relatives prior to the administration of TCZ. All patients initially received corticosteroid therapy, which failed to improve their clinical condition or prevent further deterioration due to cytokine storm. According to the 10th edition of the Diagnostic and Treatment Protocol for Novel Coronavirus Infections issued by the National Health Commission of the People’s Republic of China (PRC and Medicine, 2023), TCZ may be considered for patients with severe or critical COVID–19 if laboratory results show significantly elevated IL–6 levels. The recommended initial dose ranges from 4 to 8 mg/kg, typically administered as 400 mg diluted in 100 mL of saline and infused over the course of 1 h. If the initial dose is ineffective, the same dosage may be repeated after 12 h, not exceeding a maximum single dose of 800 mg. For a small number of patients whose symptoms do not significantly improve after two doses, and who continue to exhibit elevated IL–6 levels, a third dose may be considered based on individual clinical judgment.

In this study, the 56 patients were divided into three groups according to the number of TCZ doses received: a one–dose group, a two–dose group, and a three–dose group. All patients were administered TCZ at the standard dose of 400 mg per infusion. For patients suspected of having bacterial co–infections, based on indicators such as elevated procalcitonin or persistent fever, antibiotic therapy was initiated at the discretion of the treating physician. Prophylactic anticoagulation therapy was provided based on individual body weight and renal function. Additionally, all patients with a nucleic acid cycle threshold (CT) value below 30 received antiviral treatment.

### 2.3 Data collection

Research data were collected from the patient’s electronic medical records. For all three groups receiving different dosages of TCZ, demographic information, comorbidities, treatment regimens, in–hospital complications, and clinical outcomes were collected. In addition, laboratory test results obtained during hospitalization were recorded, including complete blood count, coagulation parameters (activated partial thromboplastin time and D–dimer), and inflammatory markers such as C–reactive protein, procalcitonin, ferritin, hyaluronic acid, and IL–6.

### 2.4 Study outcomes

Clinical parameters and inflammatory marker data were collected prior to TCZ administration and within 1 week following each dose. The primary outcome of the study was to compare in–hospital mortality and 30–day mortality following the first dose among patients with severe or critical COVID–19 receiving different dosages of TCZ. Secondary outcomes included changes in inflammatory marker levels, length of hospital stay, duration of mechanical ventilation, and the incidence of in–hospital complications, such as respiratory failure requiring mechanical ventilation, heart failure, secondary infections, thrombotic/embolic events, transaminase elevation, neutropenia, GI perforation/Haemorrhage, or acute kidney injury.

### 2.5 Statistical analysis

Statistical analyses and data visualization were performed using SPSS version 27.0 and GraphPad Prism version 9. For continuous variables, the Shapiro–Wilk test was used to assess normality. Normally distributed data were presented as mean ± standard deviation (±SD), and comparisons among groups were conducted using one–way analysis of variance (ANOVA). Non–normally distributed data were expressed as median with interquartile range (P_25_, P_75_), and group comparisons were performed using the Kruskal–Wallis H test. Categorical variables were reported as frequencies and percentages (n%), and comparisons between groups were made using the chi–square (χ^2^) test or Fisher’s exact test, as appropriate. Standardized mean difference (SMD) calculated using Cohen’s h formula. Kaplan–Meier survival analysis was used to evaluate survival outcomes, and the log–rank test was applied to compare unadjusted survival rates across treatment groups. Time–dependent COX regression was used to analyze 30–day mortality and in–hospital mortality, as well as the interaction of cortisol hormone combined with TCZ. Additionally, generalized multivariate regression analysis was used to further assess other clinical outcome variables. All statistical tests were two–sided, with a significance level set at p < 0.05.

## 3 Results

In this study, a retrospective analysis was conducted on 300 patients with severe or critical COVID–19 between January 1, 2023, and January 1, 2024, of whom 56 met the inclusion criteria and were enrolled. Among these, 16 patients (28.5%) received a single dose of TCZ, 32 patients (57.2%) received two doses, and eight patients (14.3%) received three doses. There was no significant difference in gender distribution across the three groups, and the mean age was 77.41 years (SD ± 8.98). The most common comorbidity was hypertension (60.7%), followed by cardiovascular disease (39.3%) and type 2 diabetes (14.3%). The median time from symptom onset to COVID–19 diagnosis was 7 days (interquartile range [IQR]: 6–10 days). During the disease course, 54 patients (96.4%) received antimicrobial therapy, 53 (94.6%) received anticoagulation, all 56 patients (100.0%) received corticosteroids, and 47 (83.9%) received antiviral treatment (Table 1).

**TABLE 1:** Demographic characteristics of patients treated with different doses of TCZ.

Variable	Total (n = 56)	One dose (n = 16)	Two doses (n = 32)	Three doses (n = 8)	F/H/χ^2^	P Value
Age ( $\bar{x}$ ± s, years)	77.41 ± 8.98	77.38 ± 7.40	76.00 ± 9.21	83.13 ± 9.70	2.095	0.133
Gender [n (%)] Male Female	34 (60.7%) 22 (39.3%)	10 (29.4%) 6 (27.3%)	20 (58.8%) 12 (54.5%)	4 (11.8%) 4 (18.2%)	0.556	0.860
Body Mass Index ( $\bar{x}$ ± s,kg/m^2^)	23.89 ± 3.66	24.06 ± 2.37	23.94 ± 4.36	23.34 ± 2.97	0.108	0.898
Nucleic acid positive duration (M(P_25_,P_75_), days)	15 (11.25, 20.0)	14 (11.25, 20.7)	14.5 (11.2,16.0)	18 (10.5, 29.7)	1.680	0.432
Days from onset to admission (M(P_25_,P_75_), days)	7 (6, 10)	6 (3, 10)	8 (7, 10)	7 (7, 16.7)	5.232	0.073
The time interval from symptom onset to the first use of TCZ (M(P_25_, P_75_), days)	11 (9, 15)	11 (7.5, 14)	11.5 (9, 15)	9 (8, 20)	0.892	0.640
Comorbidities [n (%)]						
Hypertension	34	7 (20.6%)	23 (67.7%)	4 (11.8%)	4.014	0.157
Diabetes	22	6 (27.3%)	14 (63.6%)	2 (9.1%)	0.917	0.645
Heart disease	8	1 (12.5%)	6 (75.0%)	1 (12.5%)	1.242	0.665
Respiratory disease	9	4 (44.4%)	4 (44.4%)	1 (11.1%)	1.383	0.602
Tumor	4	1 (25.0%)	3 (75.0%)	0 (0.0%)	0.522	1.000
Disease of the immune system	2	0 (0.0%)	2 (100.0%)	0 (0.0%)	1.035	0.668
Treatment						
Antibiotics [n (%)]	53	16 (30.2%)	30 (56.6%)	7 (13.2%)	1.883	0.387
Corticosteroids [n (%)]	56	16 (28.6%)	32 (57.1%)	8 (14.3%)	–a	–a
Anticoagulants [n (%)]	54	16 (29.6%)	30 (55.6%)	8 (14.8%)	1.035	0.668
Antiviral drugs [n (%)]	47	16 (34.0%)	24 (51.1%)	7 (14.9%)	5.039	0.066

^a^ All patients received corticosteroids, and no effective calculation was performed.

Notably, the most commonly used corticosteroid was dexamethasone sodium phosphate (n = 38), followed by methylprednisolone sodium succinate (n = 18) (Supplementary Table S1). The mean daily dose for dexamethasone was 7.32 mg (range: 5–10 mg), administered for an average duration of 6.45 days (range: 2–10 days). In contrast, methylprednisolone was given at a higher mean daily dose of 50.0 mg (range: 40–80 mg), also for approximately 6.83 days on average (range: 5–9 days).

5 patients did not require oxygen therapy at admission, but all had progressed to severe or critical illness by the time of TCZ administration (Table 2). At that point, 18 patients required non–invasive ventilation (NIV), and 4 required endotracheal intubation. High–flow nasal catheter (HFNC) use was higher in the three groups than in the one/two dose group (one dose 25.0% vs. two doses 12.5% vs. three doses 50.0%), and Mask oxygen therapy was not used in the three doses (one dose 18.7% vs two doses 40.6%). Although the p–value of the chi–square test did not reach statistical significance (HFNC: P = 0.059, Mask oxygen therapy: P = 0.052), the standardized mean difference (SMD) further supported the existence of a difference between the groups: in Mask oxygen therapy, SMD1: – 0.446, SMD2: 0.564, SMD3:0.869, in HFNC, SMD1:0.306, SMD2: −0.500, SMD3: −0.841.

**TABLE 2:** Standardized Mean Differences (SMD) for different doses of TCZ Oxygen Therapy Modalities.

Oxygen therapy methods	One–dose (n = 16)	Two–dose (n = 32)	Three–dose (n = 8)	χ^2^	P value	SMD1	SMD2	SMD3
Nasal Catheter	2 (12.5%)	3 (9.4%)	1 (12.5%)	0.565	1.000	0.104	0.0	−0.104
Mask oxygen therapy	3 (18.7%)	13 (40.6%)	0 (0.0%)	5.943	0.052	−0.446	0.564	0.869
High flow nasal cannula	4 (25.0%)	4 (12.5%)	4 (50.0%)	5.201	0.059	0.306	−0.500	−0.841
Non–invasive ventilator	7 (43.8%)	9 (28.1%)	2 (25.0%)	1.403	0.608	0.306	0.330	0.066
Endotracheal intubation	0 (0.0%)	3 (9.4%)	1 (12.5%)	1.909	0.449	−0.377	−0.645	−0.104

SMD1: one dose * two doses, SMD2: one dose * three doses, SMD3: two doses * three doses.

In addition, the clinical indications for repeated second and third doses of TCZwere analyzed. For increased oxygen demand (≥20% increase in FiO_2_ or change to advanced respiratory support): the incidence of one, two, and three doses was 31.1% (5/16), 50.0% (16/32), and 87.5% (7/8), in that order, with the Cochran–Armitage trend test showing an increasing trend (P = 0.013). For SaO_2_ decline (<90% or ≥5% from baseline): one–, two–, and three–dose incidence was 37.5% (6/16), 65.5% (21/32), and 87.5% (7/8), in that order, with a trend test of P = 0.018. Exacerbation of lung shadowing (≥50% increase in shadowing area or the development of ARDS): three doses incidence (62.5%) was though higher than one dose (50.0%), but the difference between groups was not statistically significant (trend test P = 0.678) (Table 3). In addition, baseline laboratory parameters were analyzed for patients receiving different doses of TCZ (Table 4).

**TABLE 3:** Analysis of clinical indicators and cochran–armitage trend test for different doses of TCZ.

Clinical indicators	Crude analysis			χ2	P Value	Test the trend p value
	One dose	Two doses	Three doses			
Increasing oxygen requirement rowhead	5/16 (31.1%)	16/32 (50.0%)	7/8 (87.5%)	6.750	0.034	0.013
Falling SaO2 rowhead	6/16 (37.5%)	21/32 (65.5%)	7/8 (87.5%)	6.345	0.042	0.018
Increasing pulmonary shadows rowhead	8/16 (50.0%)	15/32 (46.9%)	5/8 (62.5%)	0.625	0.732	0.678

**TABLE 4:** Baseline laboratory characteristics of patients treated with different doses of tocilizumab.

Laboratory characteristics	One dose (n = 16)	Two doses (n = 32)	Three doses (n = 8)	H	P Value
Lymphocyte count (×10^9^/L)	0.36 (0.28, 0.59)	0.62 (0.42, 0.72)	0.51 (0.23, 0.99)	4.155	0.125
Neutrophil count (×10^9^/L)	4.52 (3.73,5.99)	5.60 (4.15, 7.39)	9.82 (1.52, 18.44)	1.396	0.498
White blood cell count (×10^9^/L)	5.69 (4.62,7.69)	6.57 (4.17, 8.96)	10.90 (2.41, 19.48)	0.825	0.662
Platelet count (×10^9^/L)	167.50 (100.25, 254.75)	183.00 (136.50, 232.75)	156.00 (93.75, 196.50)	1.320	0.517
C–Reactive protein (mg/L)	27.10 (13.39, 92.41)	69.31 (30.66, 104.69)	46.02 (28.76, 65.37)	3.040	0.219
Activated partial thromboplastin time (s)	30.65 (26.62, 33.45)	30.40 (27.67, 32.02)	30.25 (28.67, 32.40)	0.585	0.746
D–Dimer (mg/L)	0.35 (0.15, 0.48)	0.32 (0.23, 0.74)	0.71 (0.25, 3.17)	2.208	0.332
Procalcitonin (ng/mL)	0.17 (0.07, 0.31)	0.13 (0.09, 0.17)	0.39 (0.13, 1.00)	4.584	0.101
Ferritin (ng/mL)	480.00 (223.00, 1412.50)	725.30 (208.75, 1105.00)	737.50 (372.50, 928.00)	0.592	0.744
Hyaluronic acid (ng/mL)	419.19 (239.36, 983.00)	353.35 (204.43, 436.76)	456.76 (172.48, 870.67)	1.986	0.371
Interleukin–6 (pg/mL)	38.70 (5.75, 52.43)	64.64 (11.08, 141.37)	44.41 (23.21, 165.06)	3.993	0.136

### 3.1 Primary outcomes

**FIGURE 1:**
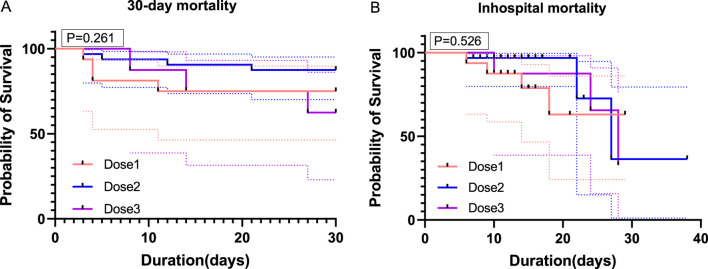
Survival curves for patients receiving different doses of TCZ. **(A)** shows the survival curves of the three groups of patients over 30 days of follow–up from the start of the first TCZ treatment. **(B)** shows the survival curves during hospitalization for the three groups of patients. On the horizontal axis, it shows time (in days), while on the vertical axis, it shows the probability of survival (in percentage). Dose1 (one–dose); Dose 2 (two–dose); Dose 3 (three–dose).

In–hospital mortality was 25.0% (4 patients) in the one–dose group, 9.3% (3 patients) in the two–dose group, and 37.5% (3 patients) in the three–dose group. The timing of TCZ administration was not standardized, and the dosing measure was modeled as a time–dependent covariate in order to eliminate the monumental time bias, and the timing of TCZ administration is shown in Supplementary Table S2. For in–hospital mortality, the unadjusted risk ratios (HRs) compared with the one–dose group showed an HR of 0.35 (95% CI: 0.15–0.80, P = 0.012) for the two–dose group and the HR for the three–dose group was 0.45 (95% CI: 0.18–1.10, P = 0.080). However, after adjusting for confounders such as baseline oxygen therapy, age, gender, BMI, disease type, duration of symptoms, interval between symptom onset and first TCZ use, comorbidities, and medication use, the adjusted HRs for the two–dose group and three–dose group were 0.65 (95% CI: 0.35–1.25, P = 0.090) and 0.70 (95% CI: 0.40–1.50, P = 0.300). Overall, the difference in in–hospital mortality between the three groups was not statistically significant. A 30–days follow–up from the first dose of TCZ showed that one patient in the two–dose group died of respiratory failure the day after discharge. Compared with the one–dose group, unadjusted HRs showed 0.37 (95% CI: 0.16–0.85, P = 0.020) for the two–dose group and 0.94 (95% CI: 0.38–2.34, P = 0.908) for the three–dose group. Adjusted HRs were 0.39 (95% CI: 0.15–1.04, P = 0.060) and 0.27 (95% CI: 0.06–1.07, P = 0.064) for the two–dose and three–dose groups, respectively. There was also no statistically significant difference in 30–day mortality between the three groups (Table 5). Kaplan–Meier survival curves for both in–hospital and 30–day follow–up periods also showed no significant differences between the groups (Figure 1).

**TABLE 5:** Time–dependent Cox regression of the main outcome.

Outcomes	Dose (vs. one dose)	Unadjusted HR (95% CI)	Unadjusted p–value	Adjusted HR (95% CI)	Adjusted p value^a^
In hospital mortality, n (%)	Two doses	0.35 (0.15–0.80)	0.012	0.65 (0.35–1.25)	0.090
	Three doses	0.45 (0.18–1.10)	0.080	0.70 (0.40–1.50)	0.300
30–day mortality, n (%)	Two doses	0.37 (0.16–0.85)	0.020	0.39 (0.15–1.04)	0.060
	Three doses	0.94 (0.38–2.34)	0.908	0.27 (0.06–1.07)	0.064

^a^ In time–dependent Cox regression, confounding factors such as baseline oxygen therapy, age, sex, BMI, disease type, duration of symptoms, interval between symptom onset and first use of TCZ, comorbidities, and drug use were included.

All patients received corticosteroids therapy, and the interaction between corticosteroids regimen and TCZ was analyzed by time–dependent Cox regression. In the univariate analysis, none of the associations of corticosteroid type, dose, and treatment duration with hospitalization outcome events were statistically significant (P > 0.05). After adjusting for confounders in the multifactorial analysis, the first two remained non–significantly associated, but corticosteroid dose approached the level of significance (HR 1.521; 95% CI 0.996–2.322, P = 0.050). In terms of interaction, the interaction terms of TCZ with corticosteroid dose (HR 1.029; 95% CI 1.002–1.056, P = 0.032), and treatment duration (HR 0.880; 95% CI 0.788–0.984, P = 0.025) were statistically significant and suggesting the presence of an interaction affecting outcome, with no significant association for the interaction term of TCZ with corticosteroid type (HR 0.580; 95% CI 0.230–1.460, P = 0.247) (Table 6).

**TABLE 6:** Time-dependent Cox regression analysis of the interaction between corticosteroids regimen and tolizumab.

Variable	Single factor analysis		Multiple factor analysis	
	HR (95% CI)	P Value	HR (95% CI)	P Value
Corticosteroid type rowhead	0.215 (0.013, 3.536)	0.282	0.022 (0.000–1.176)	0.060
Corticosteroid doses rowhead	1.311 (0.919,1.868)	0.135	1.521 (0.996–2.322)	0.052
Duration of corticosteroid therapy rowhead	0.886 (0.577,1.377)	0.592	1.205 (0.744–1.953)	0.449
TCZ*Corticosteroid type rowhead			0.580 (0.230–1.460)	0.247
TCZ*Corticosteroid doses rowhead			1.029 (1.002–1.056)	0.032
TCZ*Duration of corticosteroid therapy rowhead			0.880 (0.788–0.984)	0.025

### 3.2 Secondary outcomes

#### 3.2.1 Between–group differences in laboratory parameters following different doses of TCZ treatment

**FIGURE 2:**
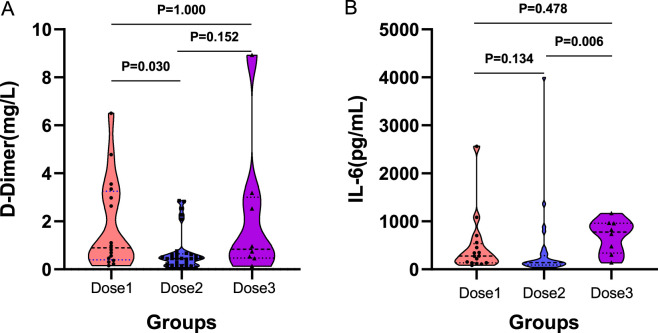
Comparison between groups after different doses of TCZ treatment. **(A)** shows the between–group differences after D–dimer treatment. **(B)** shows the between–group differences after interleukin–6 treatment. **(A)** D–Dimer; **(B)** IL–6. IL–6, Interleukin–6. Dose1 (one–dose); Dose 2 (two–dose); Dose 3 (three–dose).

Following administration of different doses of TCZ, D–dimer levels were higher in the one–dose group compared to the two–dose group (0.89 vs 0.44 mg/L, P = 0.030). However, no significant differences were observed between the one–dose and three–dose groups (0.89 vs 0.83 mg/L, P = 1.000), or between the two–dose and three–dose groups (0.44 vs 0.83 mg/L, P = 0.152) (Figure 2A). IL–6 levels were significantly elevated in the three–dose group compared to the two–dose group (775.43 vs. 138.15 pg/mL, P = 0.006). However, the differences between the one–dose and two–dose groups (278.98 vs 138.15 pg/mL, P = 0.134) and between the one–dose group and the three–dose group (278.98 vs. 775.43 pg/mL, P = 0.478) were not statistically significant (Figure 2B). In addition, no significant differences were found among the three groups in post–treatment levels of serum ferritin, hyaluronic acid, CRP, PCT, or other inflammatory markers (Supplementary Table S3).

#### 3.2.2 Changes in laboratory parameters within groups following different TCZ dosing regimens

**FIGURE 3:**
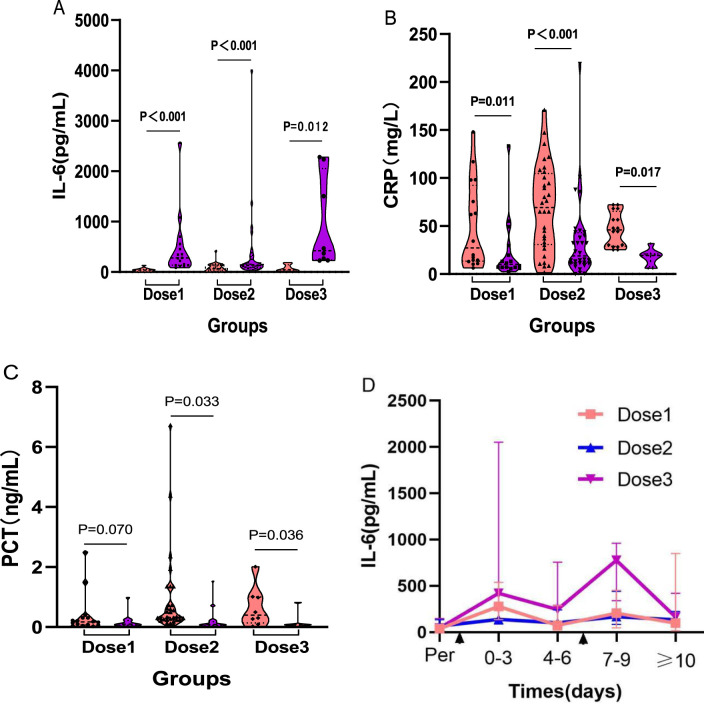
Comparison between groups before and after different doses of TCZ treatment. **(A)** shows the changes in IL–6 levels before and after TCZ treatment in the three groups. **(B)** shows the changes in CRP levels before and after TCZ treatment in the three groups. **(C)** shows the changes in PCT levels before and after TCZ treatment in the three groups. **(D)** shows the dynamic trend of IL–6 levels after TCZ administration on the horizontal axis indicates the time of TCZ administration; the first appearance represents the time of one and two doses of TCZ in the three groups of patients, and the second appearance represents the time of the third dose of TCZ in the three–dose group. Dose1 (one–dose); Dose2 (two–dose); Dose3 (three–dose).

Immediately after the completion of TCZ treatment, hematological parameters were assessed for each group using the blood test results closest to the final dose. The findings revealed that median IL–6 levels increased significantly in all three groups compared to baseline (One–dose: 38.70 vs. 278.98 pg/mL, P < 0.001; Two–dose: 64.64 vs. 138.15 pg/mL, P < 0.001; Three–dose: 44.41 vs. 775.43 pg/mL, P = 0.012) (Figure 3A). In contrast, median CRP levels decreased significantly after treatment (One–dose: 27.10 vs 10.00 mg/L, P = 0.011; Two–dose: 69.31 vs. 18.85 mg/L, P < 0.001; Three–dose: 46.02 vs 19.30 mg/L, P = 0.017) (Figure 3B). PCT levels also declined significantly in the two–dose (P = 0.033) and three–dose groups (P = 0.036), whereas the decrease in the one–dose group was not statistically significant (P = 0.070) (Figure 3C). Detailed changes in laboratory parameters before and after treatment for each group are provided in Supplementary Table S4.

To further investigate the temporal relationship between TCZ administration and IL–6 levels, a dynamic trend chart was generated (Figure 3D). In the one–dose and two–dose groups, IL–6 levels showed a transient rise within the first 0–3 days post–treatment, followed by a gradual decline. In the three–dose group, IL–6 levels followed a similar pattern after the first and second doses. However, after an initial drop, levels remained elevated. Following administration of the third dose, IL–6 levels spiked again, and although they eventually began to decline, the decrease was slower and less pronounced.

#### 3.2.3 Multivariable regression analysis of other secondary outcomes

Multivariable regression analysis was conducted to evaluate additional secondary outcomes. The average length of hospital stay for the one–dose, two–dose, and three–dose groups was 14.5, 13.0, and 18.0 days, respectively (P = 0.338), while the duration of mechanical ventilation was 10.0, 7.5, and 11.0 days, respectively (P = 0.881). Neither outcome showed statistically significant differences, and these results remained unchanged after adjusting for potential confounding variables (Table 7). Furthermore, analysis of in–hospital complications revealed no significant differences among the groups in the incidence of respiratory failure requiring mechanical ventilation, heart failure, secondary infections, thrombotic/embolic events, transaminase elevation, neutropenia, GI perforation/Haemorrhage, or acute kidney injury (Table 8).

**Figure:**
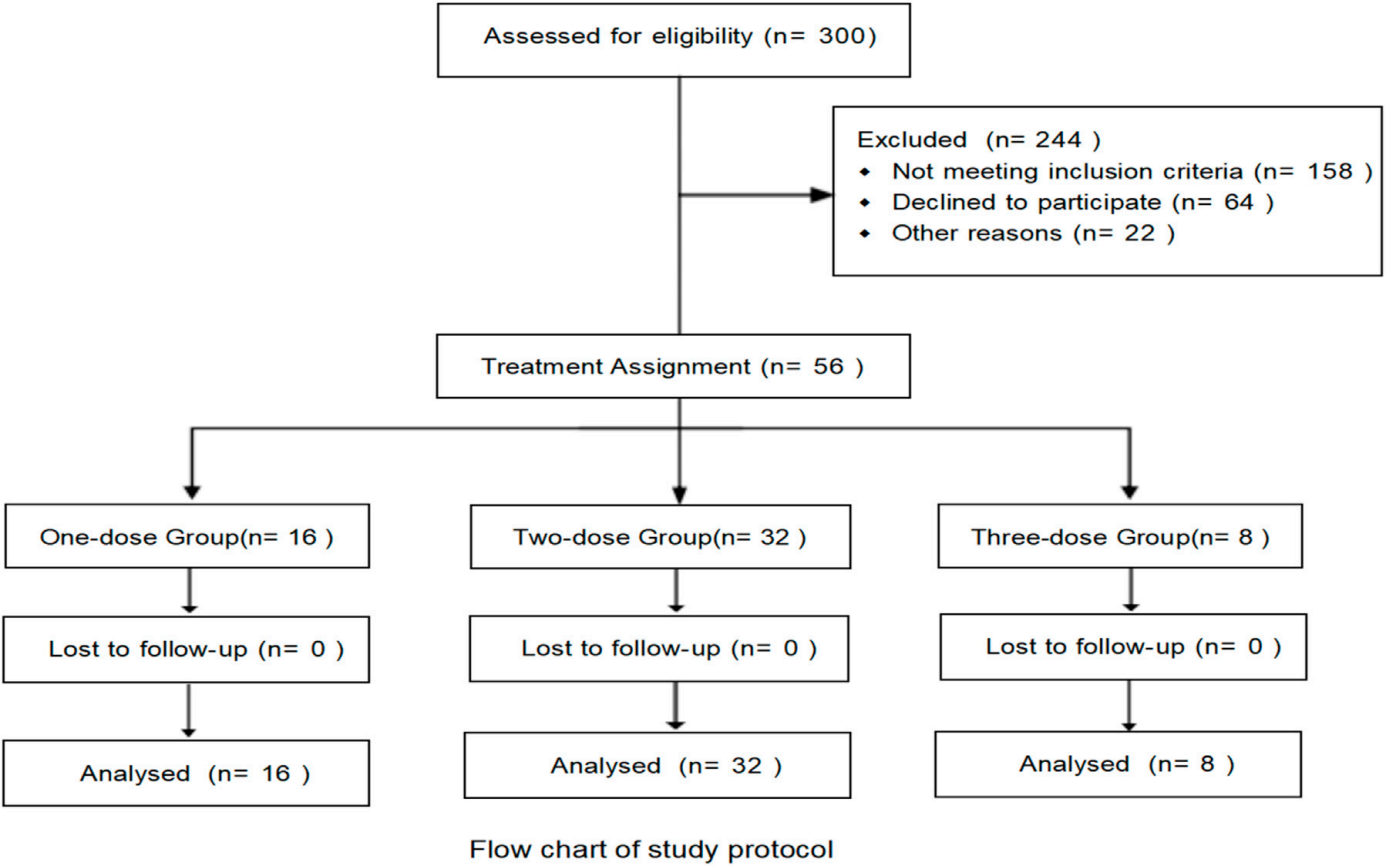


**TABLE 7:** Regression analysis of hospital length of stay and duration of mechanical ventilation.

Outcomes	Crude analysis One dose	Two doses	Three doses	H	P–value^a^	Beta coefficient (estimates) (95% CI)	P–value^b^
Length of hospitalization (M(P25,P75), days)	14.5 (11.2, 20.2)	13.0 (10.0, 16.0)	18.0 (10.5, 27.0)	2.167	0.338	1.844 (−0.950, 4.138)	0.190
Duration of mechanically assisted ventilation (M(P25,P75), days)	10.0 (8.0, 12.5)	7.5 (5.5, 12.5)	11.0 (7.0, 16.5)	0.253	0.881	−1.770 (−3.999, 0.459)	0.117

^a^ Calculate the P–value with the Kruskal–Wallis H Test.

^b^ Multivariate linear regression analysis was conducted to calculate the beta coefficients (estimates) and p–values, taking into account factors such as gender, age, BMI, disease severity, days from onset to hospital admission, Oxygen therapy methods, time interval from symptom onset to first TCZ, use, comorbidities, and medication use.

**TABLE 8:** Regression analysis of complications during hospitalization.

Outcomes	Crude analysis One dose	Two doses	Three doses	χ2	P–value^a^	Beta coefficient (estimates) (95% CI)	P–value^b^
Respiratory failure requiring mechanical ventilation n (%)	7/16 (43.8%)	12/32 (37.5%)	3/8 (37.5%)	0.187	0.911	0.673 (0.232, 1.952)	0.466
Heart failure n (%)	2/16 (12.5%)	2/32 (6.3%)	2/8 (25.0%)	2.427	0.297	1.353 (0.224, 8.175)	0.742
Secondary infections n (%)	5/16 (31.3%)	7/32 (21.9%)	2/8 (25.0%)	0.500	0.779	1.014 (0.298, 3.451)	0.982
Thrombotic/embolic events n (%)	5/16 (31.3%)	6/32 (18.8%)	2/8 (25.0%)	0.952	0.621	0.705 (0.230, 2.159)	0.540
Acute kidney injury n (%)	1/16 (6.3%)	2/32 (6.3%)	2/8 (25.0%)	2.965	0.227	2.303 (0.348, 15.220)	0.387
Transaminase elevation n (%)	8/16 (50.0%)	15/32 (46.9%)	5/8 (62.5%)	0.625	0.732	0.806 (0.261, 2.490)	0.708
Neutropenia	1/16 (6.3%)	2/32 (6.3%)	3/8 (37.5%)	7.000	0.030	1.249 (0.270,5.774)	0.776
GI perforation/Haemorrhage	1/16 (6.3%)	1/32 (3.1%)	1/8 (12.5%)	1.145	0.564	1.550 (0.222,10.837)	0.659

^a^ The chi–square test is used to calculate the P–value.

^b^ Multivariate linear regression analysis was conducted to calculate the beta coefficients (estimates) and p–valuess, including gender, age, BMI, disease severity, days from onset to hospital admission, Oxygen therapy methods, time interval from symptom onset to first TCZ, use, comorbidities, and medication use.

## 4 Discussion

This retrospective cohort study evaluated the effects of different TCZ dosing regimens (one, two, or three 400–mg doses) on clinical outcomes in patients with severe or critical COVID–19. The results of the study showed that there were differences in in–hospital mortality and 30–days mortality after the first dose of TCZ in patients with severe or critical neocoronary disease treated with one or two TCZs, but no such significant differences were observed between patients treated with one and three TCZs. Similarly, the incidence of in–hospital complications, length of stay, and duration of mechanical ventilation were comparable across the three groups. Although baseline characteristics were well balanced, adjustments were made for potential confounding factors, including the small sample size, variable dosing intervals, and the circulation of different viral variants. However, even after adjustment, patients did not show significant improvement in clinical outcomes. Notably, all groups showed a favorable response in terms of reduction in inflammatory markers.

TCZ has been shown to significantly reduce mortality in critically ill COVID–19 patients (De Roquetaillade et al., 2022; De Rossi et al., 2020; Rezaei et al., 2021), and its safety and efficacy have been validated in large–scale studies (Shankar–Hari et al., 2021). Although the therapeutic benefits of TCZ are widely recognized, the optimal dosing regimen remains a subject of debate. Guaraldi and co–workers (Guaraldi et al., 2020) suggested that critically ill patients may require a second dose to maintain adequate plasma drug concentrations, an approach supported by pharmacokinetic studies in cytokine release syndrome caused by CAR–T cell therapy (Le et al., 2018). Similarly, the tenth edition of China’s COVID–19 Diagnosis and Treatment Plan (PRC and Medicine, 2023) recommends the use of TCZ in severe and critical cases with markedly elevated IL–6 levels, allowing up to two cumulative doses. However, some studies have questioned the benefit of multiple doses. For instance, Mughal and colleagues (Mughal et al., 2020) found that administering two or more doses did not reduce all–cause mortality or improve secondary outcomes such as ICU admission, acute kidney injury (AKI), acute respiratory distress syndrome (ARDS), acute cardiac injury (ACI), thrombotic events, septic shock, or overall hospital stay. Al Sulaiman and co–workers (Al Sulaiman et al., 2021) also reported that multiple doses of TCZ did not confer additional survival benefits or reduce ICU length of stay or mechanical ventilation requirements in critically ill patients. Although the theoretical justification for repeat dosing, supported by pharmacokinetic models derived from cytokine release syndrome and national treatment guidelines such as China’s COVID–19 protocol, remains biologically plausible, our findings do not provide empirical support for its clinical efficacy in patients with COVID–19.

In contrast to our findings, large randomized controlled trials such as RECOVERY and REMAP–CAP reported significant mortality reductions with TCZ in hospitalized patients with COVID–19 (RECOVERY Collaborative Group. (2021); Gordon et al., 2021). Several factors may account for these differences. First, our cohort included a higher proportion of critically ill patients, many of whom received TCZ later in the disease course, potentially beyond the optimal therapeutic window. In RECOVERY and REMAP–CAP, earlier intervention, particularly within the hyperinflammatory phase, may have contributed to improved outcomes. Second, corticosteroid co–treatment was uniformly applied in our cohort, whereas in RECOVERY, corticosteroid use was not universal at the time of enrollment. This could have attenuated the incremental benefit of additional TCZ dosing in our study. Third, unlike these trials, our retrospective design lacked a control group that received no TCZ, limiting our ability to assess the absolute efficacy of a single dose. These differences emphasize the importance of patient selection, timing of administration, and treatment synergy in evaluating TCZ’s clinical impact.

In the present study, we found that the interaction between dose and treatment duration in the combination regimen of TCZ and corticosteroids has an impact on clinical outcomes. In terms of dose–dependent synergistic effects, corticosteroids may enhance TCZ efficacy through dual inhibition of the IL–6 pathway with systemic inflammatory responses. However, the marginal significance of the corticosteroid dose in this study (HR = 1.521, P = 0.052) also suggests the need to be alert to the potential risk of excessive immunosuppression. Notably, the negative interaction of TCZ with corticosteroid regimen suggests that early initiation of TCZ and a shorter corticosteroid regimen may optimize efficacy, consistent with the findings of the 2022 multicenter study (Jing et al. (2022)). However, the small sample size resulted in insufficient statistical power, and wide confidence intervals (e.g., the lower limit of the CI for the HR for corticosteroid dosing was close to 1.0) suggest the need for validation in larger studies. In the future, prospective designs are needed to clarify the optimal corticosteroid dose threshold and TCZ dosing timing, and to explore biomarker–guided individualized combination strategies to balance efficacy and infection risk.

In the present study, we maintained strict control over the dosage and frequency of TCZ administration. Our analysis showed that the two–dose group did not exhibit any improvement in in–hospital or 30–day mortality compared to the one–dose group. Additionally, no significant differences were found among the groups in terms of complication rates, length of hospital stay, or duration of mechanical ventilation. We also specifically evaluated the effect of a third dose to determine whether increased dosing might lead to improved clinical outcomes. Although the three–dose group did not show better outcomes compared to the one–or two–dose groups, it also did not present an increased risk of secondary infections. This contrasts with findings from Al Sulaiman et al. (2021), who reported a higher incidence of hospital–or ventilator–associated pneumonia with multiple doses. This difference may stem from multiple factors. In terms of patients’ baseline characteristics, those who received the third dose in this study had a higher oxygen demand at baseline than the other groups, and there was a difference in the choice of oxygen therapy modalities, which may have relied more on modalities such as high–flow oxygen therapy or noninvasive ventilation. Meanwhile, IL–6 levels in the three groups, although not different from the other two groups at baseline, were more significantly elevated in the third dose during follow–up, reflecting a more intense inflammatory response and greater disease severity. These factors may have acted together to cause the difference in results from other studies.

This study demonstrates that patients showed favorable changes in inflammatory markers following treatment with varying doses of TCZ. IL–6, a pivotal cytokine in the inflammatory cascade triggered by SARS–CoV–2 infection, plays a dual role, exhibiting both pro–and anti–inflammatory effects, and is critically involved in disease progression (Fajgenbaum and June, 2020). IL–6 signals through two pathways: classical signaling via membrane–bound IL–6 receptors (mIL–6R) and trans–signaling via soluble IL–6 receptors (sIL–6R). While low serum IL–6 levels are associated with anti–inflammatory responses, elevated IL–6 levels promote a pro–inflammatory state through trans–signaling, thereby activating a broader range of cells (Wang et al., 2022). This dysregulation is a hallmark of the hyperinflammatory response observed in severe COVID–19.

In this study, all three treatment groups exhibited increases in IL–6 levels following TCZ administration, typically showing a transient spike followed by a gradual decline with some fluctuation, a trend consistent with findings from a recent study by Liang and colleagues (Liang et al., 2024). This pattern aligns with TCZ’s mechanism of action: by inhibiting IL–6 receptors, it prevents IL–6 from binding, leading to an accumulation of unbound IL–6 in the serum (Killer et al., 2024). Notably, Mughal and colleagues (Mughal et al., 2020) reported no significant difference in post–treatment peak IL–6 levels between patients receiving a single dose versus multiple doses. Similarly, our study found comparable IL–6 levels after treatment in the one–dose and two–dose groups. However, patients in the three–dose group exhibited a more pronounced increase in IL–6 levels after treatment. This could be attributed to a cumulative pharmacological effect, individual variability in immune responses, or a more intense cytokine storm in these patients.

Simultaneously, this study found no statistically significant differences in CRP levels among the three TCZ treatment groups, which are consistent with findings from previous studies (Mughal et al., 2020). This suggests that the extent of CRP reduction may not be dependent on the number of TCZ doses administered. CRP levels decreased in all three groups after treatment compared to baseline, indicating that TCZ contributed to effective inflammation control (Calderon–Ochoa et al., 2024). In prior follow–up studies, patients receiving multiple doses of TCZ exhibited higher post–treatment PCT levels, which were associated with an increased risk of hospital–or ventilator–acquired infections (Al Sulaiman et al., 2021). However, in the present study, the risk of secondary infections did not appear to rise with additional doses of TCZ, and no significant differences in PCT levels were observed among the three groups after treatment. The variations in post–treatment PCT levels relative to baseline may reflect the influence of other endogenous anti–inflammatory factors, synergistic immune regulatory mechanisms, and individual patient variability.

Although different dosing regimens of TCZ demonstrated some positive effects on inflammatory markers, these improvements did not translate into reduced mortality or better clinical outcomes in critically ill COVID–19 patients. However, there were limitations in this study, with small and unbalanced sample sizes in the three groups (n = 16/32/8), resulting in the study lacking sufficient statistical power to detect moderate effect sizes, and upon *post hoc* efficacy analyses, the statistical power of the moderate differences in 30–day deaths and hospitalized deaths was found to be approximately 39.8%–41.2% and 38.7%–42.1%, which is well below the conventional threshold of 80%. This further confirms the lack of statistical validity. Thus, no firm conclusions can be drawn regarding the dose–response relationship based on the statistical power limitations of the current study. Also, the clinical benefit of the second dose is questionable due to the limitations of the current study, although the limited sample size may be one of the main influencing factors. In addition, there were deficiencies in safety reporting. Although no increase in secondary infections was observed between dose groups, this finding needs to be interpreted with caution given the incomplete surveillance of infections and the lack of detailed follow–up of adverse events.

This study has several limitations that warrant consideration. First, its retrospective, single–center design and small, imbalanced sample size (n = 16/32/8 across groups) limit generalizability and reduce statistical power to detect moderate effect sizes, as reflected in the wide confidence intervals observed for key outcomes. Accordingly, the findings should be interpreted as exploratory and hypothesis–generating rather than definitive. Second, the timing and frequency of TCZ administration were not standardized and were left to physician discretion, introducing potential for confounding by indication and immortal–time bias. Although time–dependent COX regression analyses were performed, fewer outcome events occurred before the typical window period for tolizumab administration, which influenced the impact of immortalization time on our specific cohort. Third, baseline severity may have varied between groups, especially in terms of oxygen treatment modality, oxygen demand, and SaO2, as echoed in our data, which may have led to bias in the comparison of treatment response. Fourth, subgroup analyses of type, dose, and duration of corticosteroids were not performed, and specific cutoff values for the effect on outcomes were not obtained, which affected the analysis of temporal synergism between corticosteroids and TCZ. Finally, although adverse events have been refined, the timing of their occurrence is difficult to obtain, the temporal association between multiple TCZ and complications is difficult to effectively address, and future prospective studies need to evaluate safety outcomes more systematically.

## 5 Conclusion

In this study of patients with severe and critical COVID–19, no additional benefit was observed with further dosing; larger studies are needed to confirm this finding. Due to the lack of a control group that did not receive TCZ, the efficacy of a single dose cannot be determined. These findings highlight the need for careful consideration of individual patient characteristics when determining dosing strategies.

## Data Availability

The original contributions presented in the study are included in the article/Supplementary Material, further inquiries can be directed to the corresponding authors.
