# Supplementary material for: Evaluating the efficacy of different doses of tocilizumab in treating critically ill COVID–19 patients: a single–center retrospective cohort study
Source: Front Pharmacol. 2025 Jul 24;16:1571372. doi: 10.3389/fphar.2025.1571372 (PMC12328359; doi:10.3389/fphar.2025.1571372)
Supplement: Supplementary file 1 [file DataSheet1.docx]

Supplementary Material

# Supplementary Data

Supplementary Table 1. Timing, type, and duration of corticosteroid co-treatment in patients receiving tocilizumab.

| Corticosteroid type | N | Mean dose (mg) | Dose range | Mean duration (days) | Duration range |
| --- | --- | --- | --- | --- | --- |
| Dexamethasone sodium phosphate | 38 | 7.32 | 5 – 10 | 6.45 | 2 – 10 |
| Methylprednisolone sodium succinate | 18 | 50.00 | 40 – 80 | 6.83 | 5 – 9 |

Supplementary Table 2.Different TCZ dosing intervals were used

|  | One-dose  （n=16） | Two-dose  （n=32) | Three-dose  （n=8） | H/Z | P value |
| --- | --- | --- | --- | --- | --- |
| The time interval from symptom onset to the first use of TCZ ( M（P_25_, P_75_）, days) | 11（7, 14） | 11（9, 15） | 9（8, 20） | 0.892 | 0.640 |
| Time interval between first and second TCZ use (M (P25, P75), days) | - | 1（1, 1） | 1（1, 1） | -0.555 | 0.579 |
| Time of second-third dose (M (P25, P75), days) |  |  | 6（4,7） |  |  |

Supplementary Table 3. Between-group changes in laboratory parameters after different doses of tocilizumab treatment.

|  | One-dose  （n=16） | Two-dose  （n=32) | Three-dose  （n=8） | H | P value |
| --- | --- | --- | --- | --- | --- |
| Lymphocyte Count (×10^9^/L) | 0.51（0.30，1.00） | 0.67（0.37，0.91） | 0.43（0.32，0.93） | 0.810 | 0.667 |
| Neutrophil Count (×10^9^/L) | 7.70（3.99，11.57） | 6.31（4.56，8.36） | 7.72（4.50，11.41） | 1.089 | 0.580 |
| White Blood Cell Count (×10^9^/L) | 8.68（5.82，11.99） | 7.25（5.49，10.46） | 8.80（5.24，12.45） | 1.323 | 0.516 |
| Platelet Count (×10^9^/L) | 204.50（146.25，262.00） | 201.00（155.50，259.50） | 153.00（114.25，212.75） | 2.105 | 0.349 |
| C-Reactive Protein (mg/L) | 10.00（6.93，27.23） | 18.85（11.42，42.98） | 23.67（20.92，34.37） | 4.354 | 0.113 |
| Activated Partial  Thromboplastin Time (s) | 28.6（25.2，30.82） | 27.90（26.37，30.45） | 31.50（26.10，34.44） | 1.449 | 0.485 |
| D-Dimer (mg/L) | 0.89（0.39，3.25） | 0.44（0.19，0.64） | 0.83（0.46，3.00） | 8.397 | 0.015 |
| Procalcitonin (ng/mL) | 0.09（0.06，0.21） | 0.08（0.05，0.17） | 0.11（0.05，0.37） | 1.603 | 0.449 |
| Ferritin (ng/mL) | 578.00（349.00，1020.00） | 679.70（303.00，986.00） | 495.00（375.25，1084.25） | 0.003 | 0.998 |
| Hyaluronic Acid (ng/mL) | 488.67（237.96，966.63） | 358.08（163.54，858.11） | 477.42（202.91，936.30） | 1.965 | 0.374 |
| Interleukin-6 (pg/mL) | 278.98（135.98，535.92） | 138.15（89.39，285.10） | 775.43（340.16,958.76） | 11.160 | 0.004 |

D-Dimer: Does 1-Does 2: P=0.030; Does 1-Does 3: P=1.000; Does 2-Does 3: P=0.152;

IL-6: Does 1-Does 2: P=0.134; Does 1-Does 3: P=0.478; Does 2-Does 3: P=0.006;

Supplementary Table 4. Changes in laboratory parameters in the group after different doses of tocilizumab treatment.

|  | pretherapy | post-treatment | Z | P value |
| --- | --- | --- | --- | --- |
| One-dose  Lymphocyte Count (×109/L)  Neutrophil Count (×109/L)  White Blood Cell Count (×109/L)  Platelet Count (×109/L)  C-Reactive Protein (mg/L)  Activated Partial Thromboplastin Time (s)  D-Dimer (mg/L)  Procalcitonin (ng/mL)  Ferritin (ng/mL)  Hyaluronic Acid (ng/mL)  Interleukin-6 (pg/mL) | 0.36（0.28，0.59）  4.52（3.73，5.99）  5.69（4.62，7.69）  167.50（100.25，254.75）  27.10（13.39，92.41）  30.65（26.62，33.45）  0.35（0.15，0.48）  0.17（0.07，0.31）  480.00（223.00，1412.50）  419.19（239.36，983.00）  38.70（6.02，52.43） | 0.51（0.30，1.00）  7.70（3.99，11.57）  8.68（5.82，11.99）  204.50（146.25，262.00）  10.00（6.93，27.23）  28.6（25.2，30.82）  0.89（0.39，3.25）  0.09（0.06，0.21）  578.00（349.00，1020.00）  488.67（237.96，966.63）  278.98（135.98，535.92） | -1.678  -2.844  -3.051  -1.810  -2.534  -1.966  -2.792  -1.810  -0.973  -0.078  -3.516 | 0.093  0.004  0.002  0.070  0.011  0.049  0.005  0.070  0.330  0.937  ＜0.001 |
| Two-dose  Lymphocyte Count (×109/L)  Neutrophil Count (×109/L)  White Blood Cell Count (×109/L)  Platelet Count (×109/L)  C-Reactive Protein (mg/L)  Activated Partial Thromboplastin Time (s)  D-Dimer (mg/L)  Procalcitonin (ng/mL)  Ferritin (ng/mL)  Hyaluronic Acid (ng/mL)  Interleukin-6 (pg/mL) | 0.62（0.42，0.72）  5.60（4.15，7.39）  6.57（4.17，8.96）  183.00（136.50，232.75）  69.31（30.66，104.69）  30.40（27.67，32.02）  0.32（0.23，0.74）  0.13（0.09，0.17）  725.30（208.75，1105.00）  353.35（204.43，436.76）  64.64(11.08，159.33) | 0.67（0.37，0.91）  6.31（4.56，8.36）  7.25（5.49，10.46）  201.00（155.50，259.50）  18.85（11.42，42.98）  27.90（26.37，30.45）  0.44（0.19，0.64）  0.08（0.05，0.17）  679.70（303.00，986.00）  358.08（163.54，858.11）  138.15（89.39，285.10） | -1.178  -0.813  -1.206  -3.160  -3.684  -1.973  -0.262  -2.132  -0.430  -1.085  -3.609 | 0.239  0.416  0.228  0.002  ＜0.001  0.049  0.793  0.033  0.667  0.278  ＜0.001 |
| Three-dose  Lymphocyte Count (×109/L)  Neutrophil Count (×109/L)  White Blood Cell Count (×109/L)  Platelet Count (×109/L)  C-Reactive Protein (mg/L)  Activated Partial Thromboplastin Time (s)  D-Dimer (mg/L)  Procalcitonin (ng/mL)  Ferritin (ng/mL)  Hyaluronic Acid (ng/mL)  Interleukin-6 (pg/mL) | 0.51（0.23，0.99）  9.82（1.52，18.44）  10.90（2.41，19.48）  156.00（93.75，196.50）  46.02（28.76，65.37）  30.25（28.67，32.40）  0.71（0.25，3.17）  0.39（0.13，1.00）  737.50（372.50，928.00）  456.76（172.48，870.67）  44.41（22.84，146.28） | 0.86（0.53，1.20）  8.59（4.83，12.91）  10.55（6.65，14.33）  173.00（118.75，237.75）  19.30（7.89，21.03）  31.75（30.44，37.57）  0.59（0.34，0.94）  0.07（0.03，0.09）  472.50（358.50，1026.00）  260.27（153.64，862.30）  775.43（340.16，958.76） | -1.540  -0.420  -0.140  -0.560  -2.380  -0.700  -0.980  -2.100  -0.420  -0.338  -2.521 | 0.123  0.674  0.889  0.575  0.017  0.484  0.327  0.036  0.674  0.735  0.012 |
